# Variation between seated and standing/walking postures among male and female call centre operators

**DOI:** 10.1186/1471-2458-12-154

**Published:** 2012-03-02

**Authors:** Allan Toomingas, Mikael Forsman, Svend Erik Mathiassen, Marina Heiden, Tohr Nilsson

**Affiliations:** 1 Karolinska Institutet, Institute of Environmental Medicine, Stockholm, Sweden; 2 Centre for Musculoskeletal Research, Department of Occupational and Public Health Sciences, University of Gävle, Gävle, Sweden; 3 Karolinska Institutet, Department of Public Health Sciences, Stockholm, Sweden; 4 University of Umeå, Department of Public Health and Clinical Medicine, Umeå, Sweden

## Abstract

**Background:**

The dose and time-pattern of sitting has been suggested in public health research to be an important determinant of risk for developing a number of diseases, including cardiovascular disorders and diabetes. The aim of the present study was to assess the time-pattern of seated and standing/walking postures amongst male and female call centre operators, on the basis of whole-shift posture recordings, analysed and described by a number of novel variables describing posture variation.

**Methods:**

Seated vs. standing/walking was recorded using dichotomous inclinometers throughout an entire work shift for 43 male and 97 female call centre operators at 16 call centres. Data were analysed using an extensive set of variables describing occurrence of and switches between seated and standing/walking, posture similarity across the day, and compliance with standard recommendations for computer work.

**Results:**

The majority of the operators, both male and female, spent more than 80% of the shift in a seated posture with an average of 10.4 switches/hour between seated and standing/walking or vice versa. Females spent, on average, 11% of the day in periods of sustained sitting longer than 1 hour; males 4.6% (p = 0.013). Only 38% and 11% of the operators complied with standard recommendations of getting an uninterrupted break from seated posture of at least 5 or 10 minutes, respectively, within each hour of work. Two thirds of all investigated variables showed coefficients of variation between subjects above 0.5. Since work tasks and contractual break schedules were observed to be essentially similar across operators and across days, this indicates that sedentary behaviours differed substantially between individuals.

**Conclusions:**

The extensive occurrence of uninterrupted seated work indicates that efforts should be made at call centres - and probably in other settings in the office sector - to introduce more physical variation in terms of standing/walking periods during the work day. We suggest the metrics used in this study for quantifying variation in sedentary behaviour to be of interest even for other dichotomous exposures relevant to occupational and public health, for instance physical activity/inactivity.

## Background

Computer work dominates occupational life in many countries worldwide, e.g. within the European Union [1]. Typically, 70-75% of the workforce use computers in their jobs [2]. Computer work is usually performed in a seated posture. Therefore, jobs characterized by extensive use of computers are likely to imply extensive periods of sitting and low levels of energy expenditure.

Computers particularly dominate work at call centres (CC), or *Contact centres, Customer contact centres *or *Customer support centres*, as they are increasingly termed. CCs employ 2-3% of the work force in many Western countries, and have been claimed to be one of the fastest growing trade sectors since their introduction on a larger scale in the mid 1990ies [3]. In 2005, the number of CC agents was estimated to be approximately 4 million in the US, 200,000 in France, 300,000 in Germany, 800,000 in the UK, and 100,000 in Sweden [4]. Work in CCs has been described as sedentary, repetitive and monotonous, both mentally and physically [3]. However, CC work varies in mental complexity from short cycled repetitive operations, such as ticket booking and phone-number information, to complicated and intellectually demanding tasks such as financial or medical advice and computer support. The common denominator for all CC work is the total dominance of customer communication by telephone, assisted by computers. Thus, CC work is mostly performed while seated at a computer workstation [5].

Office work comprising extensive computer use has been suggested to be a risk factor for musculoskeletal disorders, and this apprehension has been supported by several studies [6-12], while others have been less conclusive [13,14].

A growing concern has, however, been raised about the possible health effects of extensive sitting at offices and in other occupations beyond possible musculoskeletal risks. Sedentary behaviour per se is known from public health research to be associated with a range of serious health risks, including obesity, hypertension, type II diabetes, metabolic syndrome, venous thromboembolism, cardiovascular diseases, cancer and also increased mortality [15-24]. These risks are associated with sitting per se, to a large extent independent of whether the individual is otherwise physically active [17]. Since most people between 18 and 65 years of age spend a large part of their wake time at work, and since several occupations has been shown to imply extensive sitting [25], sitting in occupational life may contribute significantly to the negative public health effects of sedentariness.

Traditional office ergonomics has primarily focused risk reduction strategies on decreasing the amplitude of muscle and joint loads, but recent studies have stressed the importance of focussing on temporal aspects of work, i.e. the exposure variation [21,26,27]. In general, ample variation in physical exposure has been proposed to be a major prerequisite for good musculoskeletal health [28,29]. This focus on the time-line of exposure rather than just the exposure level is well in line with recent public health research on sedentariness, suggesting that interventions should not only address the total duration of sitting, but also the time distribution of breaks from sedentary time [30].

In a typical office setting comprising extensive seated computer work, physical variation can be achieved through gross body movements, including leaving the work station. Ideally, this variation would occur naturally if the job per se contained tasks offering (non-seated) exposures differing markedly from those associated with computer work [31]. In CC settings, such "productive variation" is not easy to create, since very little core CC work can be done away from the computer workstation. The major feasible option for obtaining variation is therefore to change between seated and standing work-postures at the computer. Other, more infrequent opportunities are to leave the computer for coffee and lunch breaks, visit the rest-room, or attend to staff-meetings.

In spite of the increasing awareness among researchers and practitioners that physical variation is important, even in terms of breaking up extensive sedentary periods, only a few studies have been devoted to variation in physical workload among office workers performing their usual work. Juul-Kristensen & Jensen [32] classified variation among office workers in broad categories, but did not collect any quantitative data. Other studies report postural variation during computer work, either in the field [33,34] or during controlled tasks in the laboratory [27,35,36], but these studies have concentrated on arm and neck postures. The occurrence of seated (sedentary) and non-seated work among office or computer workers has been addressed in only a few studies, using either self-reported time of sitting [37,38] or inclinometry [39,40]. None of these studies assessed the time-pattern of seated and non-seated postures.

One reason that variation in occupational loads and postures has rarely been studied in quantitative terms is probably the paucity of standardized methods to measure "variation" [28,29]. Interestingly, a similar frustration in public health research recently led to the proposal of a generic technique to characterize the distribution of sedentary behaviour, based on mathematical modelling [41]. Conceptually, "variation" in any physical exposure has been suggested to include three different aspects [28]: A) *How much *does exposure change; B) *How fast (or how often) *does exposure change; C) *How similar *are exposure periods.

Ergonomic recommendations for variation in office work combine these aspects, in particular 'how much' and 'how often', in stating that workers should leave their computer workstation for at least 5 to 10 minutes every hour [42-45]. The intention of this recommendation is to give the worker an adequate break, both from a likely sedentary posture, from a steady muscular load with little variation in the shoulder region and the arms, and from the mental demands of the dominating work tasks. None of the recommendations state explicitly whether breaks from seated should be uninterrupted or not. Thus, both an uninterrupted 5-10 minute period in a non-seated posture and shorter but more frequent breaks adding up to 5-10 minutes every hour would satisfy the recommendations. While previous research have indicated that compliance can be a critical concern in implementing initiatives related to break behaviour, no systematic investigation has yet been made of whether office workers behave according to recommendations or not.

Possible differences in the sedentary behaviour of males and females are of interest in this context, since female professional computer users, including CC operators, report more symptoms and other health-related problems than male professional computer users [11,46]. The long-term metabolic health effects of sedentary behaviour also seem to differ between men and women [16,20].

The aim of this study was to assess variation in gross body postures amongst male and female CC operators on the basis of whole-shift recordings of seated and standing/walking. To serve this purpose, the paper introduces a number of novel variables describing the temporal structure of postural behaviour. The following aspects were explicitly posed, analysing gender differences: posture duration, frequency of posture changes, similarities in the posture pattern across time, and compliance with standard recommendations for variation.

## Methods

### Study design, call centres and subjects

The present investigation utilized data from a comprehensive cross-sectional study conducted in 2002-03 in Sweden, examining working conditions and health among employees at CCs [47]. Sixteen CCs were included: six "internal" customer support departments within a parent company, and 10 "external" enterprises giving customer support to other companies. Four of the CCs were public and 12 private companies; 11 had national and five international owners. The CCs were located in both urban (seven CCs) and rural areas (nine CCs). All CCs gave customer support at different levels of complexity. Details of the main study design, including a closer description of the participating CCs, have been reported elsewhere [5,48].

Ten operators on duty on the days of study were randomly selected at each of the 16 CCs. Four operators refused to participate, and thus 156 operators (47 male; 109 female) were included.

The study was approved by the Ethical committee at the Karolinska Institutet (No: 01-332).

### Posture measurements

Whole-shift recordings of seated versus standing/walking postures were collected at a dichotomous level using a portable inclinometer with a data logger (weight 375 gram) (Posimeter 100, Biolin AB, Mölndal, Sweden) (Additional file 1: Figure A1) The inclinometer sensor was attached to the lateral side of the right thigh in the sagittal plane and registered a "seated" posture when the angle of the thigh was less than 45° from horizontal and a "standing/walking" posture when the angle was 45° or more. According to previous studies the inclinometer discriminated between sitting and standing/walking with a satisfying validity and reliability [49]. Notably, the inclinometer did not discriminate between standing and walking, and so a non-seated posture is described as "standing/walking" in the present paper. Subjects were informed that the device recorded the posture of their leg.

The inclinometer sampled postures at a frequency of three Hz. A software filter was set to ignore periods shorter than three seconds in order to avoid artifacts from shakings, walking on stairs etc. The duration, in seconds, of each separate period of seated or standing/walking was logged in real-time. The posture recordings began when the shift commenced and were stopped at the end of the shift. Thus, the recordings included coffee-breaks, lunch, etc. Ordinary shifts lasted eight hours.

### Data processing and analysis

All 156 recordings were visually inspected as a quality control. Sixteen recordings were rejected due to technical failures of the inclinometer, leaving 140 whole-shift recordings (43 male and 97 female) for further analysis. Four categories of variables describing exposure level and aspects of exposure variation were addressed (Table 1). A simulated recording is provided in the (Additional file 1: Figure A2) to illustrate each of the variables defined in Table 1. The variables were calculated in Microsoft Excel, version 2003. Product moment correlations were calculated for selected pairs of variables. Differences between male and female operators were tested using Student's two-tailed *t*-test, unless the distribution of a variable deviated significantly from normal according to a Kolmogorov-Smirnov Z test (PASW Statistics 18; SPSS Inc., Chicago, IL, USA), in which case the Mann-Whitney test was used. Differences in the proportions of males and females complying with the standard recommendations for variation in office work were tested using a two-sample proportion test (Epicalc 2000, vers. 1.02). Eight different versions of criteria were tested. P-values < 0.05 were regarded as statistically significant.

**Table 1:** Definitions of variables describing the occurrence and variation of postures in the present study.

Category & name	Definition
**Exposure level**	
**LEV1**	Proportion of total time in seated posture (%)
**Frequency**	
**FREQ1**	Frequency of switches from seated to standing/walking or *vice-versa *(h^-1^)
**FREQ2**	Average duration of uninterrupted periods seated (min)
**FREQ3**	Average duration of uninterrupted periods standing/walking (min)
**FREQ4**	Proportion of total time in uninterrupted periods seated longer than one hour (%)
**FREQ5**	Proportion of total time in uninterrupted periods standing/walking longer than one hour (%)
**Similarity**	
**SIM1**	Coefficient of variation (SD/mean); durations of periods seated
**SIM2**	Coefficient of variation (SD/mean); durations of periods standing/walking
**SIM3**	Correlation between the duration of periods seated and the following period standing/walking^a^
**Criteria basis for compliance with guidelines**	
**CRIT1**	Average time until 5 minutes of standing/walking have been accumulated (min)^a; b^
**CRIT2**	Average time until 5 minutes of uninterrupted standing/walking have been obtained (min)^a; b^
**CRIT3**	Average time until 10 minutes of standing/walking have been accumulated (min)^a; b^
**CRIT4**	Average time until 10 minutes of uninterrupted standing/walking have been obtained (min)^a; b^
**CRIT5**	Proportion of total time when standing/walking has not occurred for 5 accumulated minutes during the previous hour (%)^c^
**CRIT6**	Proportion of total time when standing/walking has not occurred for 5 uninterrupted minutes during the previous hour (%)^c^
**CRIT7**	Proportion of total time when standing/walking has not occurred for 10 accumulated minutes during the previous hour (%)^c^
**CRIT8**	Proportion of total time when standing/walking has not occurred for 10 uninterrupted minutes during the previous hour (%)^c^

^a ^Analysis begins by first period seated, disregarding any possible standing/walking period in the very beginning of the recording.

^b ^When a period containing the necessary standing/walking time has been obtained, time count restarts at the next following occurrence of sitting.

^c ^Exposure time-line analysed using a 60-minute moving window at one second increments. Ten minutes preceding the start of the actual recording was considered to be standing/walking, since the recording equipment was attached while the subject was standing.

## Results

The 140 operators included in the analyses had an average age of 34 years and mean seniority at the company 3.5 years (Additional file 1: Table A1). The 16 rejected had a mean age of 34 years; mean seniority 1.4 years, and four of them were males. Women were on average about 5 years older and had one year longer seniority than the men, who were taller and heavier, as expected, but also had a higher BMI (Additional file 1: Table A1). A BMI ≥25 kg/m^2^, defined as above normal [50,51], was noted for 36% of the operators (males 53%; females 29%).

In total, 817 hours were recorded (males 254; females 563). The average recording time for an operator was 5.8 hours (males 5.9, females 5.8; Table 2). Examples of recordings are shown in the (Additional file 1: Figure A3). Shift durations differed depending on rosters applied, and because some operators worked part-time or overtime. In total, 8575 separate posture periods were recorded (males 2989; females 5586), out of which 4218 were seated (1473; 2745) and 4357 standing/walking (1516; 2841). As the recordings usually started and ended with a standing posture, the number of standing periods generally was one larger than the number of seated periods.

**Table 2:** Total recording time and variables describing exposure levels and frequency of changes in exposure as defined in Table 1; in total and amongst male and female call centre operators.

				Difference males-females	
**Variable**	**All** **N = 140**	**Males** **N = 43**	**Females** **N = 97**	**Difference**	**p-value**^**1**^
**Recording time**	**Total recording time (hours)**				
**Mean**	5.84	5.91	5.81	0.09	0.650
**Median**	5.98	6.02	5.93	0.09	
**sd**	1.13	1.25	1.07		
**Range**	1.49-8.83	1.49-8.83	3.00-8.54		
**LEV1**	**Proportion in seated posture (%)**				
**Mean**	74.9	74.1	75.3	-1.1	0.477*
**Median**	80.6	80.5	80.8	-0.3	
**sd**	16.6	17.3	16.3		
**Range**	6.2-95.4	6.2-93.3	15.9-95.4		
**FREQ1**	**Frequency of posture switches (per hour)**				
**Mean**	10.4	11.5	9.9	1.5	0.090
**Median**	9.9	11.3	8.8	2.5	
**sd**	4.9	4.7	4.9		
**Range**	2.5-25.4	2.5-21.2	3.2-25.4		
**FREQ2**	**Average duration of seated periods (min)**				
**Mean**	11.2	9.8	11.8	-1.9	0.114
**Median**	9.9	8.7	10.5	-1.8	
**sd**	6.7	6.5	6.7		
**Range**	0.5-39.5	0.5-39.5	1.9-33.8		
**FREQ3**	**Average duration of standing/walking periods (min)**				
**Mean**	3.1	2.8	3.3	-0.5	0.498*
**Median**	2.6	2.3	2.6	-0.3	
**sd**	2.3	1.7	2.6		
**Range**	0.8-18.7	0.9-8.0	0.8-18.7		
**FREQ4**	**Per cent of time in uninterrupted seated periods > 1 hour**				
**Mean**	9.1	4.6	11.1	-6.6	0.013*
**Median**	0.0	0.0	0.0	0.0	
**sd**	15.7	11.4	16.9		
**Range**	0.0-78.8	0.0-43.9	0.0-78.8		
**FREQ5**	**Per cent of time in uninterrupted stand/walk periods > 1 hour**				
**Mean**	1.9	1.5	2.0	-0.6	0.386*
**Median**	0.0	0.0	0.0	0.0	
**sd**	6.6	6.9	6.5		
**Range**	0.0-36.5	0.0-36.5	0.0-27.7		

^1 ^Two-tailed t-tests or Mann-Whitney test (*) if significant deviation from normal distribution. Boldface p-values indicate a significant difference (p < 0.05).

### Overall posture proportions

**Figure 1:**
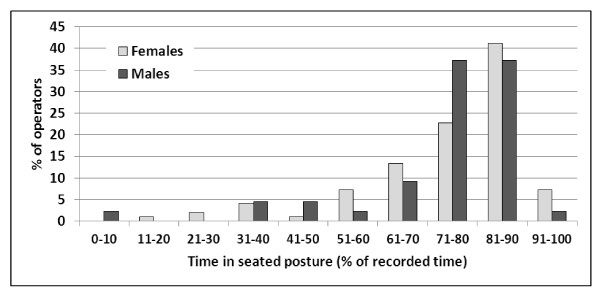
**Distribution of the proportion of the work shift spent in a seated posture amongst male (n = 43) and female (n = 97) call centre operators**.

On average, 75% (median 81%) of the recorded time was spent seated (LEV1) with a large range (6-95%) among operators (Table 2, Figure 1). No significant difference was observed between males and females. Total shift duration and per cent time in seated did not correlate (product moment r = -0.0001).

### Frequency of posture changes

**Figure 2:**
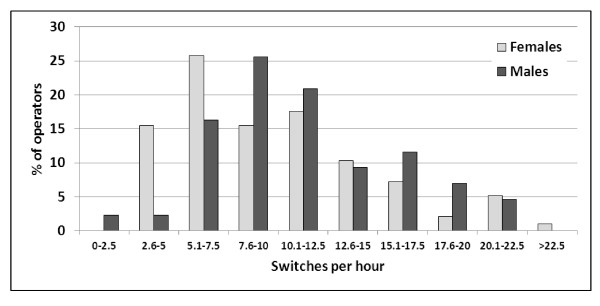
**Distribution of the number of switches per hour from seated to standing/walking posture or vice versa among male (n = 43) and female (n = 97) call centre operators**.

The average frequency of switches from seated to standing/walking or vice versa (FREQ1) was 10.4 per hour, females having on average 9.9 shifts per hour and males 11.5 (p = 0.090) (Table 2; Figure 2). The average duration of a seated period (FREQ2) was 11.2 minutes, and a standing/walking period (FREQ3) was 3.1 minutes. Females tended to have somewhat, while not significantly, longer periods, both in seated and standing/walking.

**Figure 3:**
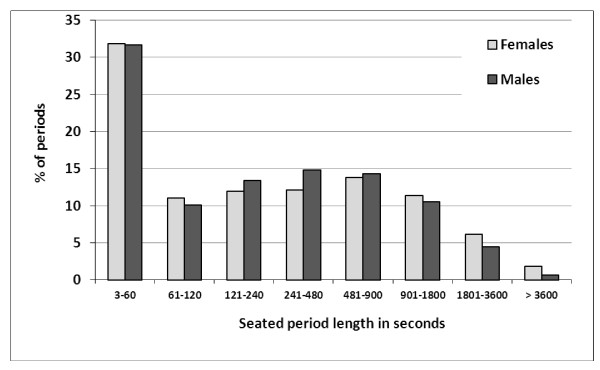
**Proportion (in%) of seated periods (males n = 1473, females n = 2745) according to duration amongst male (n = 43) and female (n = 97) call centre operators**.

**Figure 4:**
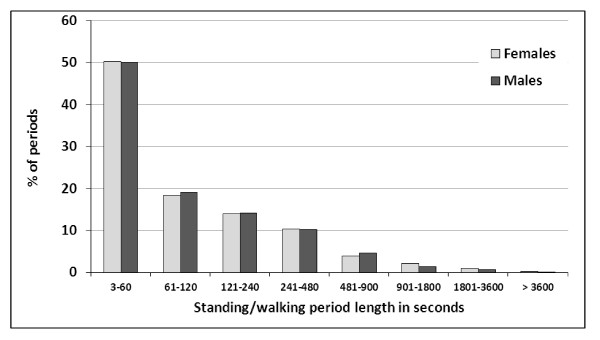
**Proportion (in%) of standing/walking periods (males n = 1516, females n = 2841) according to duration amongst male (n = 43) and female (n = 97) call centre operators**.

The distributions of the duration of seated and standing/walking periods were asymmetric (Figures 3 and 4). Approximately one third of all seated periods and half of all standing periods lasted one minute or less. A sub-classification of all periods of up to 100 seconds revealed a peak occurrence of periods lasting 6-10 seconds for both seated and standing/walking postures (Additional file 1: Figures A4 and Additional file 1: Figures A5). All 1045 seated and standing/walking periods lasting 3-10 seconds, i.e. 12.2% of all 8575 recorded periods, were analysed in detail. Out of these, 74 were pairs of two consecutive periods both being 3-10 seconds long, 19 were triplets, three quadruplets and one comprised six consecutive periods. All other periods, i.e. 826 (79%) of the 1045 periods, were single 3-10 second periods surrounded by periods longer than 10 seconds.

**Figure 5:**
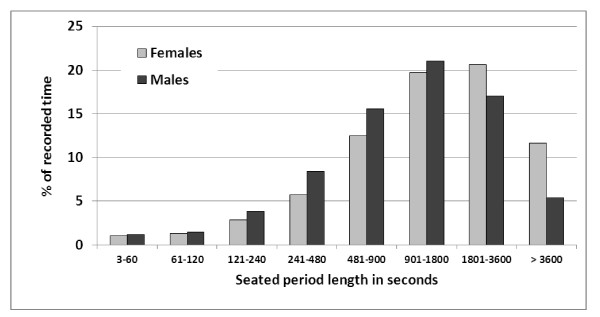
**Proportion (in%) of total recording time (males 3993 min; females 8323 min) spent in seated periods of different period lengths amongst male (n = 43) and female (n = 97) call centre operators**.

**Figure 6:**
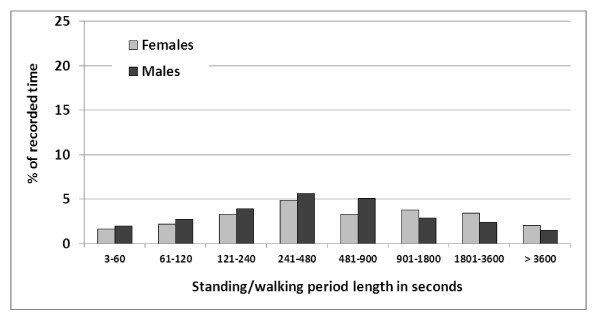
**Proportion (in%) of total recording time (males 3993 min; females 8323 min) spent in standing/walking periods of different period lengths amongst male (n = 43) and female (n = 97) call centre operators**.

The distribution of periods of different durations, expressed in terms of the proportion of total time spent seated or standing/walking, respectively, was fairly symmetric when plotted on a "logarithmic" scale (Figures 5 and 6). About 20% of all recorded time was spent in seated periods lasting 15-30 minutes and another 20% in seated periods lasting 30-60 minutes. On the average, 9.1% of the recorded time was spent in uninterrupted seated periods exceeding one hour (FREQ4; Table 2). Females spent a significantly higher proportion of time in these "long" periods (11.0% versus 4.6%). For both sexes, less than 2% of the total time was spent standing/walking in uninterrupted periods exceeding one hour (FREQ5). Large inter-individual differences were noted, in particular for the proportion of total time in uninterrupted periods seated (FREQ4; range 0%-79%; CV 1.7) and standing/walking (FREQ5; range 0%-37%; CV 3.5) longer than one hour. CV = Coefficient of Variation (the standard deviation divided by the mean); in these cases indicating a substantial variation in relation to the mean values.

### Similarity of postures

The mean coefficient of variation for the duration of single periods of seated (SIM1) and standing/walking postures (SIM2) was between 1.3 and 1.5 (Table 3). The average correlation between durations of consecutive seated and standing periods (SIM3) was 0.07. No significant differences were noted between males and females. Thus, durations of seated and standing periods varied considerably, yet not in any regular pattern.

**Table 3:** Outcome in variables describing similarity of exposure across time as defined in Table 1; in total and amongst male and female call centre operators.

				Difference males-females	
**Variable**	**All** **N = 140**	**Males** **N = 43**	**Females** **N = 97**	**Difference**	**p-value**^**1**^
**SIM1**	**Coefficient of variation within individuals; duration of seated periods**				
**Mean**	1.31	1.26	1.33	-0.07	0.212
**Median**	1.24	1.21	1.26	-0.05	
**sd**	0.32	0.31	0.32		
**Range**	0.69-2.34	0.70-2.09	0.69-2.34		
**SIM2**	**Coefficient of variation within individuals; duration of standing/walking periods**				
**Mean**	1.51	1.50	1.51	-0.01	0.759*
**Median**	1.34	1.35	1.32	0.03	
**sd**	0.55	0.49	0.57		
**Range**	0.58-3.37	0.64-2.85	0.58-3.37		
**SIM3**	**Correlation between the duration of periods seated and the following standing/walking period**				
**Mean**	0.07	0.07	0.08	-0.01	0.762
**Median**	0.04	0.04	0.06	-0.02	
**sd**	0.22	0.22	0.22		
**Range**	-0.42 - 1.00	-0.26 - 1.00	-0.42 - 0.85		

^1^Two-tailed t-tests or Mann-Whitney test (*) if significant deviation from normal distribution.

### Compliance with recommendations

On average, 43 minutes passed until 5 minutes of standing/walking had been accumulated (CRIT1; Table 1). This increased to 69 minutes when an accumulated 10 minutes of standing/walking was required (CRIT3). Adopting the stricter criterion of obtaining an *uninterrupted *period of 5 or 10 minutes of standing/walking (CRIT2 and CRIT4) substantially increased these durations to 98 minutes and 204 minutes, respectively. Female operators took longer time to obtain the recommended variation, and the gender difference was significant for the time passing until an accumulation of 10 minutes standing/walking (CRIT3). The proportion of operators who, as an average throughout the shift, complied with obtaining sufficient variation within 60 minutes varied between 11% and 84% depending on the criterion (COMPLYcrit1 - COMPLYcrit4; Table 5). For three of the four criteria, more males than females complied, and the difference was significant for 5 minutes accumulated standing/walking (COMPLYcrit1).

**Table 4:** Outcome in variables describing compliance with standard recommendations for variation in computer work (CRIT1 - CRIT8) as defined in Table 1; in total and amongst male and female call centre operators.

				Difference males-females	
**Variable**	**All** **N = 140**	**Males** **N = 43**	**Females** **N = 97**	**Difference**	**p-value**^**1**^
**CRIT1**	**Duration until accumulation of 5 minutes of standing/walking (min)**				
**Mean**	43.1	37.8	45.5	-7.7	0.057
**Median**	40.5	36.5	41.6	-5.1	
**sd**	21.9	16.3	23.8		
**Range**	6.6-154	6.6-88.6	8.8-154		
**CRIT2**	**Duration until obtaining an uninterrupted period of 5 minutes in standing/walking (min)**				
**Mean**	97.8	85.3	103	-18.1	0.619*
**Median**	75.3	72.1	77.1	-5.0	
**sd**	84.4	64.6	91.6		
**Range**	9.7-469	9.7-400	11.4-469		
**CRIT3**	**Duration until accumulation of 10 minutes of standing/walking (min)**				
**Mean**	68.7	60.1	72.5	-12.4	**0.048**
**Median**	63.2	61.5	65.0	-3.5	
**sd**	34.2	23.3	37.5		
**Range**	12.0-240	12.0-133	15.7-240		
**CRIT4**	**Duration until obtaining an uninterrupted period of 10 minutes in standing/walking (min)**				
**Mean**	204	192	209	-17	0.474*
**Median**	184	175	188	-13	
**sd**	122	120	123		
**Range**	18.6-491	18.6-453	25.5-491		
**CRIT5**	**Proportion of time not standing/walking for 5 accumulated minutes during the previous 60 minutes (%)**				
**Mean**	19.0	15.9	20.4	-4.6	0.251*
**Median**	13.4	11.0	15.0	-4.1	
**sd**	17.6	14.3	18.7		
**Range**	0.0-80.6	0.0-57.2	0.0-80.6		
**CRIT6**	**Proportion of time not standing/walking for an uninterrupted 5 minute period during the previous 60 minutes (%)**				
**Mean**	39.5	37.8	40.2	-2.4	0.576
**Median**	40.1	39.0	40.4	-1.4	
**sd**	23.3	21.1	24.2		
**Range**	0.0-88.2	0.0-86.2	0.0-88.2		
**CRIT7**	**Proportion of time not standing/walking for 10 accumulated minutes during the previous 60 minutes (%)**				
**Mean**	40.0	36.8	41.5	-4.7	0.277
**Median**	41.6	38.3	45.5	-7.1	
**sd**	23.7	20.4	24.9		
**Range**	0.0-86.4	0.0-73.2	0.0-86.4		
**CRIT8**	**Proportion of time not standing/walking for an uninterrupted 10 minute period during the previous 60 minutes (%)**				
**Mean**	64.7	64.0	65.1	-1.1	0.709*
**Median**	71.9	71.8	72.0	-0.2	
**sd**	22.2	22.1	22.4		
**Range**	2.2-89.9	2.2-88.8	6.6-89.9		

^1 ^Two-tailed t-tests or Mann-Whitney test (*) if significant deviation from normal distribution. Boldface p-values indicate a significant difference (p < 0.05)

**Table 5:** Proportion (in%) operators complying with standard recommendations for variation in computer work (CRIT1 - CRIT8) as defined in Table 1; in total and amongst male and female call centre operators.

				Difference males-females	
**Variable**	**All** **N = 140**	**Males** **N = 43**	**Females** **N = 97**	**Difference**	**p-value**^**1**^
**COMPLYcrit1**	**On average, ≤ 60 minutes until accumulation of 5 minutes of standing/walking**				
**%**	84.3	95.4	79.4	16.0	**0.032**
**COMPLYcrit2**	**On average, ≤ 60 minutes until obtaining an *uninterrupted *period of 5 minutes in standing/walking**				
**%**	37.9	32.6	40.2	-7.7	0.502
**COMPLYcrit3**	**On average, ≤ 60 minutes until accumulation of 10 minutes of standing/walking**				
**%**	42.1	44.2	41.2	3.0	0.888
**COMPLYcrit4**	**On average, ≤ 60 minutes until obtaining an *uninterrupted *period of 10 minutes in standing/walking**				
**%**	11.4	16.3	9.3	7.0	0.361
**COMPLYcrit5**	**Has accumulated 5 minutes of standing/walking within any 60 minutes period throughout the day**				
**%**	15.7	14.0	16.5	-2.5	0.897
**COMPLYcrit6**	**Has been standing/walking for 5 minutes *without interruption *within any 60 minutes period throughout the day**				
**%**	6.4	4.7	7.2	-2.6	0.844
**COMPLYcrit7**	**Has accumulated 10 minutes of standing/walking within any 60 minutes period throughout the day**				
**%**	10.0	9.3	10.3	-1.0	0.903
**COMPLYcrit8**	**Has been standing/walking for 10 minutes *without interruption *within any 60 minutes period throughout the day**				
**%**	0.0	0.0	0.0	0.0	-

Based on data in Table 4

^1 ^Two-sample proportion test. Boldface p-values indicate a significant difference (p < 0.05)

The average proportion of total recorded time when 5 minutes of standing/walking were *not *accumulated during the preceding 60 minutes (CRIT5) was 19%, increasing to 40% if 10 minutes was required (CRIT7; Table 4). The corresponding proportions when using the stricter criterion of *uninterrupted *standing/walking periods were 40% (5 minute periods (CRIT6)) and 65% (10 minute periods (CRIT8)). These proportions were slightly, but not significantly, higher among females. The proportion of operators complying with each of these criteria throughout the entire recording period varied between 0% and 16% (COMPLYcrit5 - COMPLYcrit8; Table 5). Only minor gender differences were noted.

## Discussion

### Extensive seated work

In this cross-sectional study we assessed gross body postures by continuous real-time dichotomous recordings of seated and standing/walking amongst operators performing their routine call centre work including having breaks of different kinds. We found that the operators spent, in median, 81% of the work shift in a seated posture (LEV1). This corresponds well with the operators' own estimations of time spent seated [52]. The operators also reported that, on average, 67% of the work shift was devoted to communication with the customers, 23% was used for administrative purposes, and 10% for other duties such as staff meetings [47]. Thus the majority of the customer and administrative duties were done in a seated posture. Per cent time seated did not correlate with the total recorded time, which indicates that operators working longer shifts did not compensate their extended work with a higher proportion of standing/walking activities. Neither does it appear likely that the operators were more seated due to fatigue towards the end of long shifts.

The inclinometer could not discriminate between standing and walking. We did not use step counters or accelerometers [53], which could have resolved this issue. Neither do we have any direct information about the nature of the activities during the different seated or standing/walking periods. And we do not know whether they were performed at or away from the computer workstation. We hypothesize that part of the standing/walking time was spent at the workstation performing ordinary work tasks at a hoisted desk. This indicates that the proportion of time spent off the computer was lower than suggested by the standing/walking data. On the other hand, an unknown proportion of the seated time was spent at the coffee and lunch table (typically 2 × 15 minutes and 30 minutes respectively) and at staff meetings. This indicates an opposite bias of more time being spent off the computer than suggested by the proportion of time standing/walking. While these concerns relate to the validity of our inclinometer data as a proxy for work contents and physical exercise, they do not question that our data correctly reflect sedentary behaviours among the CC operators. Further clarifications of the nature and time-line of physical exposure during extended office and/or computer work could be obtained from studies assessing the extent of physical (metabolic) activity, while also observing actual activities occurring during periods of seated and standing/walking.

The extensive time spent seated at work in the present study corresponds well with a few other reports of seated time amongst administrators, clerks or professional computer users [25,37,39]. In an inclinometer study of CC operators, Mork and Westgaard [40] found that the operators were seated for 86% of the workday. This was similar to help-desk workers (83%), but somewhat higher than secretaries (76%). Their sample size was fairly small, however; 26 subjects in total. A study of 89 female medical secretaries using computers found that 68% of the working day (lunch excluded) was spent in a seated posture [54]. That study used the same type of inclinometer as the present study. An additional sample of 90 females and 96 males was examined in that same study (ages 16-64 years; mainly administrative, commercial, technical, healthcare or manufacturing professions). In this group, females worked seated for 45% of the day and males for 49%. Thus, secretaries using computers spent more time in a seated posture than the other occupational groups and the CC operators in the present study spent even more time in a seated posture than the secretaries.

Extensive seated work has been associated with several potentially serious health problems, mainly of a cardiovascular and metabolic nature [15,17,18,20,22-24]. Studies of CC operators with a general health focus are, however, rare [55]. While the proportion of operators in our study with an BMI above normal (25 kg/m^2^) was about the same as in the general Swedish population in the same age groups [56], 68% of the operators in a large new US CC company reported that they had gained weight since the start of their employment eight months earlier; on the average 7.5 kg, i.e. 0.9 kg/month [57]. This roughly corresponds to the expected increase in body weight from the decrease in metabolism accompanying a change from standing/walking work at 3-4 MET (Metabolic Unit; 1 MET = 1 kcal/kg/hour) to a predominantly seated work at 1.5-2 MET if other relevant factors remain unchanged, for instance energy intake. The response rate among the 1100 approached operators was, however, low: only 36% completed the survey.

### Variation - changing between seated and standing/walking postures

The number of switches from seated to standing/walking postures and vice versa was, on average, about 10 per hour in the present study (FREQ1), corresponding to about 5 periods of seated work, which lasted, on average, 11.2 minutes each (FREQ2). A large proportion, 12.2%, of all seated and standing/walking periods were of a very short duration; 10 seconds or less. No systematic observations of the work were made, so the exact nature of the activities during these short periods of seated and/or standing is unknown. Since the majority of these short periods appeared as single events, they are probably not an artefact of repetitive movements, e.g. fidgeting.

Mork & Westgaard [40] reported an average seated period duration of 14.8 minutes amongst CC operators. The average duration of periods in an upright posture was 2.2 minutes, which is similar to our findings. The inclinometer study mentioned above [54] recorded a frequency of 19 switches per hour between seated and standing/walking postures among female medical secretaries, 25 switches among the female samples and 22 switches among the males. Thus, secretaries using computers made fewer switches than the other occupational groups and the CC operators in the present study made even fewer than the secretaries.

The present study found a 10-fold dispersion between operators in the frequency of switches, indicating a large difference in working technique between operators and/or between days for the individual operator (cf. Additional file 1: Figure A3). Only a small percentage of the seated periods lasted one hour or more, but they accounted for almost 10% of the total recorded time (FREQ4). Large differences between the CC operators were found also for this variable, again indicating differences in working behaviour. The variability between operators could also be the result of differences in the contents of work, even if the CC tasks appeared very similar across workers and companies, or differences in work station design, e.g. whether sit-to-stand desks were available or not.

### Similarity

The duration of seated and standing/walking periods varied substantially *within *individual operators, as judged from the coefficient of variation (SIM1; SIM2). This indicates an irregularity in the time-pattern of change between seated and standing postures, which was further confirmed by the lack of correlation between the duration of neighbouring periods (SIM3); long seated periods were not systematically compensated by long standing/walking periods. No other study has, to our knowledge, presented a comparable data set on similarity as a descriptor of variation in professional computer work, or other occupations.

### Compliance with recommendations for computer work

Current standard recommendations for computer-based office work - which are not specifically based on metabolic concerns - advocate to have a break from computer tasks for 5-10 minutes every hour, as mentioned earlier. The findings in our study indicate that these recommendations were followed to a differing extent depending on how the recommendations are interpreted. A substantial proportion of the operators, 84% and 42% respectively, complied with the criteria of working, on average, no more than 60 minutes until 5 or 10 minutes *accumulation *had occurred (COMPLYcrit1 and COMPLYcrit3; Table 5). But only a modest proportion of the operators, 38% and 11% respectively, complied with the criteria of working, on average, no more than 60 minutes until a period of 5 or 10 minutes *uninterrupted *standing/walking had occurred (COMPLYcrit2 and COMPLYcrit4; Table 5). The analysis of consecutive 60 minute windows throughout the shift showed that such a strict interpretation of the recommendations (5-10 minutes *accumulated *or *uninterrupted *non-seated posture during *every *60 minute period - COMPLYcrit5 to COMPLYcrit8; Table 5), made compliance critical.

Our data suggest that some of these criteria for sufficient posture variation may, indeed, be very strict, and it would be interesting to obtain corresponding data from occupations with more diverse tasks for comparison. It is also important to note that our data do not indicate whether the operator actually left the computer or stopped working while in the standing/walking posture. Thus the standing/walking periods reported in the current study do represent breaks from the seated posture, but may not represent a true 'break' from the work tasks, which is also an intention in the recommendations.

The CC operators in the current study estimated that, on average, about four hours passed before they left their computer workstation for a period of 10 minutes or longer [52]. Obviously, CC operators in this study were well aware that they spent a substantial part of their shift seated. In a recent Swedish national survey, 46% of professional computer users reported that they spend less than one hour at the computer before having a break of at least 10 minutes [2]. However, 27% reported working for 1-2 hours uninterruptedly before taking a 10-minutes break, 20% worked 2-4 hours and 7% worked 4 hours or more. These statistics are in agreement with our results, corroborating that a large proportion of professional computer workers seem not to comply with the recommendation of 5-10 minutes break from a seated posture every hour, and that very long uninterrupted periods of seated postures occur to a considerable extent.

When asked by an ergonomist about important aspects of healthy computer work, 65% of the CC operators in the present study population (males 53%; females 70%) mentioned "variation of postures", while 74% mentioned the importance of "regular breaks" and "leaving the computer work-station" (males 60%; females 79%) [58]. Thus, the lack of compliance with current recommendations observed among the CC operators occurred even though they were generally aware of the benefits of variation and breaks, and 70% were equipped with a sit-stand work station [5].

When the operators were asked by the doctor doing a health check-up to rate their own risk of contracting a health problem of any kind caused by their work, the risk was estimated to be, on average, 53% (males 46%; females 56%) [58]. This prognosis is close to the actual prevalence of musculoskeletal health problems in the present population - 50-60% [46]. This might indicate that the operators mainly associate to musculoskeletal disorders when asked about health risks, and less to the risks of contracting metabolic and cardiovascular disorders due to too much sitting. This is confirmed by the answers to the doctor's question what long-term health-risks from their present job they knew of. No one mentioned cardiovascular or metabolic health problems. Pain in different body regions, eye-strain, headaches and stress-related problems were mentioned by most operators.

The fact that the operators fail to comply with recommendations known to them suggests that other drivers may override the motivation to follow these recommendations, and lead to extended sedentary behaviours. Identifying these drivers, whether at the level of the individual, the work task, or the organisation, is an important issue in the further development of effective interventions against sedentariness and lack of physical activity.

The necessary extent and structure of physical variation for reducing health risks due to long-lasting sedentariness is not known at present [28], even if breaks as short as one minute in sitting time during waking hours have been shown to restrain metabolic syndrome [24,30]. If this applies to CC-work, the large occurrence of standing/walking periods one minute or shorter among the CC operators in this study - more than 50% of all standing/walking periods - would be important to health.

None of the recommendations state explicitly whether breaks from seated work should be uninterrupted or not. Thus, both an uninterrupted 5-10 minute period in a non-seated posture and "short but numerous" breaks every hour would be recommendable according to the recommendations. Neither is the purpose of the recommendations explained in physiologic, medical or psychological terms, beyond general statements such as "the body needs variation". Their scientific basis is not explicitly stated either, even though an implicit underpinning may be found in studies showing positive effects on fatigue, discomfort and performance of allowing an additional 2-10 minute rest break every hour [59-61]. The acute effects of short and long breaks on fatigue and discomfort have been compared in a few controlled studies, which do not provide any clear answer of which is preferable [62,63]. However, long-term effects on these outcomes, let alone pain or metabolic regulation are, to our knowledge not elucidated.

### Differences between male and female operators

Compared to male operators, females spent longer uninterrupted periods in seated postures, had fewer switches between seated and standing/walking postures, and spent a longer time seated before standing/walking for a minimum of 5 or 10 minutes. Since males and females spent roughly the same proportion of the total work-day seated, female operators therefore had less variation in gross postural behaviour during their work shifts than male operators. Since males and females performed similar tasks at the studied CCs, the differences in their posture time-lines might reflect a difference in working behaviour. Whether this gender difference in sedentary behaviour has any health consequences in the present CC population is not clear. Studies have observed negative health consequences of sedentary work among both men and women. Gender differences have been observed, yet without any obvious explanation [16,20]. Besides being a concern on its own, the extended seated periods amongst women could be interpreted as a proxy for longer periods of uninterrupted activity in the neck and shoulder muscles. Gender differences in gross body posture variation could therefore be part of the explanation why female professional computer users consistently report a higher prevalence and incidence of musculoskeletal disorders than males [11,46,64].

### Implications for the future

Work with computers has come to dominate modern work life. Sedentary postures occur extensively during computer work, as demonstrated by this and other studies. Further clarifications of the nature and time-line of physical exposure during extended office work, including computerized tasks, should be obtained from studies assessing the extent of physical (metabolic) activity, while also observing the activities associated with periods of seated and standing/walking.

The growing concern that a sedentary lifestyle - including both work and leisure - is a health risk [19,21,65,66] motivates the notion that occupational work including extended computer use represents an important public health issue, both in its own right and due to its effects on leisure time activities [67]. Whether this is, indeed, the case, and what would then be appropriate initiatives to combat sedentary behaviours are important issues for future research. While being a powerful contributor to risks following from extended sitting, work may also be viewed as a possible arena for implementing public health interventions promoting less sedentariness. To this end, studies identifying effective interventions to prevent sedentary work and lack of postural variation are needed. So far, the effectiveness is not known of moderate interventions like software programs stimulating breaks [68]. Another option for obtaining breaks from sitting is to offer the computer user a workstation allowing changes between seated and standing working postures, and encourage the use of this opportunity, both by securing easy adjustment of the workstation and by educating and motivating the worker to adopt a working technique with regular switches between seated and standing. While not firmly based on specific empirical studies [28], it is a reasonable assumption that it would be beneficial to health if work at CCs were organized so that sedentary tasks are mixed with physically more active, non-seated tasks. For example, office workers could engage in organizing coffee-breaks or other activities for their team. A more advanced example would be that office workers also did the cleaning at the office. However, such alternative tasks are probably too few in current CCs to be a feasible and effective source of variation. Thus, a job enlargement initiative could be extended to include other companies outside the CC that offer alternative, diverging work tasks.

In order to understand and efficiently investigate the possible effects of initiatives like this, determinants of sedentary behaviour and lack of posture variation need be identified, and accompanied by standardized metrics of variation that reflect the relevant characteristics of these determinants. The set of metrics suggested in the present paper can serve as an inspiration, not only for studies of sedentariness, but also for other dichotomous expressions of exposure such as physical activity/inactivity and the presence or absence of activity in muscles suspected to be sensitive to extended loading [69-71].

## Conclusions

Work at the studied call-centres was dominated by extended periods in a seated posture interspaced with, to a major extent, short periods of standing/walking. While the overall proportion of seated did not differ between men and women, the average individual period of uninterrupted seated work was longer for women. Most CC operators did not comply with standard recommendations of having a 5-10 minute break from the seated posture every hour, especially if they are understood as *uninterrupted *breaks. Interventions against extensive sedentariness should focus on introducing work tasks that must be performed standing or walking. This would probably increase variation even in other biomechanical exposures.

We believe that the concepts and metrics presented in this study can be of general interest in occupational and public health research addressing time patterns of dichotomous exposures, for instance activity/inactivity during work and leisure. Longitudinal studies are needed to examine the performance and predictive validity of different metrics, as well as the significance of the extent and structure of sitting on metabolism, weight and health.

## Competing interests

The authors declare that they have no competing interests.

## Authors' contributions

AT: project leader, project conception and design, data acquisition, conceptual development of variables, analysis and interpretation of data, drafting and critically revising the manuscript. MF: conceptual development of variables, analysis and interpretation of data, critically revising the manuscript. SEM: conceptual development of variables, interpretation of data, drafting and critically revising the manuscript. MH: analysis and interpretation of data, critically revising the manuscript. TN: project conception and design, data acquisition, interpretation of data, critically revising the manuscript. All authors read and approved the final manuscript.

## Pre-publication history

The pre-publication history for this paper can be accessed here:

http://www.biomedcentral.com/1471-2458/12/154/prepub

## Supplementary Material

Additional file 1 **Figure A1 Portable inclinometer with sensor attached to the thigh**. **Figure A2 **Illustration of the calculations of variables used in the present study (cf. Table 1). An imaginary recording sequence lasting 180 minutes comprised of seated and standing/walking periods is shown. The corresponding values of the variables in Table 1 are given below the figure. **Figure A3 **Examples of recordings from three call centre operators (A-C) showing different patterns of switches between seated (0) and standing/walking (1) postures. The x-axis shows duration of recording from start (hours: minutes). **Figure A4 **Seated periods of 3-100 seconds duration in proportion (%) of the total duration all 4218 recorded seated periods, stratified by period length, amongst call centre operators observed during whole work-shifts. Detail of Figure **3 **in main file. **Figure A5 **Standing/walking periods of 3-100 seconds duration in proportion (%) of the total duration all 4357 recorded standing/walking periods, stratified by period length, amongst call centre operators observed during whole work-shifts. Detail of Figure **4 **in main file. **Table A1 **Self-reported age, seniority at present company, height, weight and calculated body mass index (BMI) for the CC operators studied at the 16 call centres; in total and amongst male and female call centre operators. Click here for file
