# Supplementary material for: Variation between seated and standing/walking postures among male and female call centre operators
Source: BMC Public Health. 2012 Mar 2;12:154. doi: 10.1186/1471-2458-12-154 (PMC3348085; doi:10.1186/1471-2458-12-154)
Supplement: Additional file 1 — Figure A1 Portable inclinometer with sensor attached to the thigh. Figure A2 Illustration of the calculations of variables used in the present study (cf. Table 1). An imaginary recording sequence lasting 180 minutes comprised of seated and standing/walking periods is shown. The corresponding values of the variables in Table 1 are given below the figure. Figure A3 Examples of recordings from three call centre operators (A-C) showing different patterns of switches between seated (0) and standing/walking (1) postures. The x-axis shows duration of recording from start (hours: minutes). Figure A4 Seated periods of 3-100 seconds duration in proportion (%) of the total duration all 4218 recorded seated periods, stratified by period length, amongst call centre operators observed during whole work-shifts. Detail of Figure 3 in main file. Figure A5 Standing/walking periods of 3-100 seconds duration in proportion (%) of the total duration all 4357 recorded standing/walking periods, stratified by period length, amongst call centre operators observed during whole work-shifts. Detail of Figure 4 in main file. Table A1 Self-reported age, seniority at present company, height, weight and calculated body mass index (BMI) for the CC operators studied at the 16 call centres; in total and amongst male and female call centre operators. [file 1471-2458-12-154-S1.DOC]

**Additional file**

*
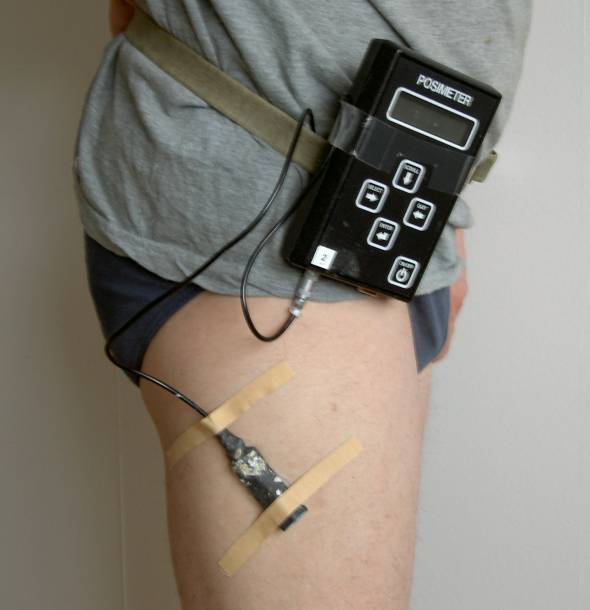
*

**Figure A1** Portable inclinometer with sensor attached to the thigh.

Time (min)

Seated

Standing/

walking

30

120

0

60

180

150

90

15

70

30

22

18

2

15

5

3

| Variable | Value | Comment |
| --- | --- | --- |
| LEV1 | 76.1 % |  |
| FREQ1 | 2.7 h^-1^ | Eight shifts in three hours |
| FREQ2 | 34.3 min | Four seated periods |
| FREQ3 | 8.6 min | 5 standing/walking periods |
| FREQ4 | 38.9 % | One period seated longer than one hour (70 minutes) |
| FREQ5 | 0.0 % | No periods standing/walking longer than one hour |
| SIM1 | 0.72 |  |
| SIM2 | 0.86 |  |
| SIM3 | 0.37 | Four sets of seated and following standing/walking periods |
| CRIT1 | 51.7 min | Three sequences: minutes 4-23, 24-98, 109-165 |
| CRIT2 | 52.3 min | Three sequences: minutes 4-23, 24-98, 109-167 |
| CRIT3 | 80.0 min | Two sequences: minutes 4-98, 109-170 |
| CRIT4 | 83.5 min | Two sequences: minutes 4-103, 109-172 |
| CRIT5 | 11.1 % | One sequence: minutes 79-98 |
| CRIT6 | 13.3 % | Two sequences: minutes 79-98, 164-166 |
| CRIT7 | 32.2 % | Two sequences: minutes 56-103, 160-169 |
| CRIT8 | 37.2 % | Two sequences: minutes 51-103, 159-172 |

**Figure A2** Illustration of the calculations of variables used in the present study (cf. Table 1). An imaginary recording sequence lasting 180 minutes comprised of seated and standing/walking periods is shown. The corresponding values of the variables in table 1 are given below the figure.


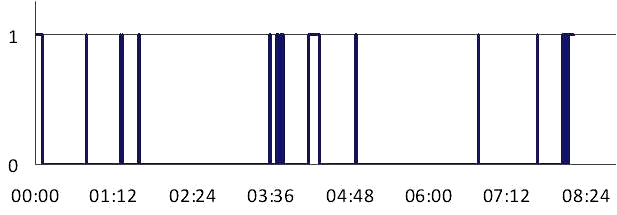


**A**

*
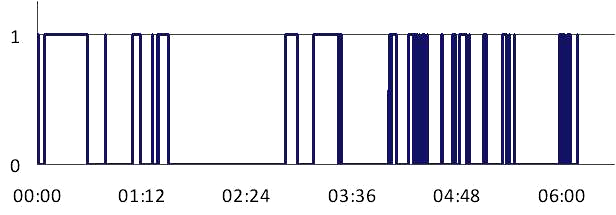
*

**B**

*
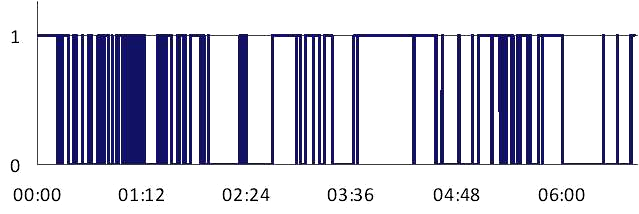
*

**C**

**Figure A3** Examples of recordings from three call centre operators (A-C) showing different patterns of switches between seated (0) and standing/walking (1) postures. The x-axis shows duration of recording from start (hours: minutes).


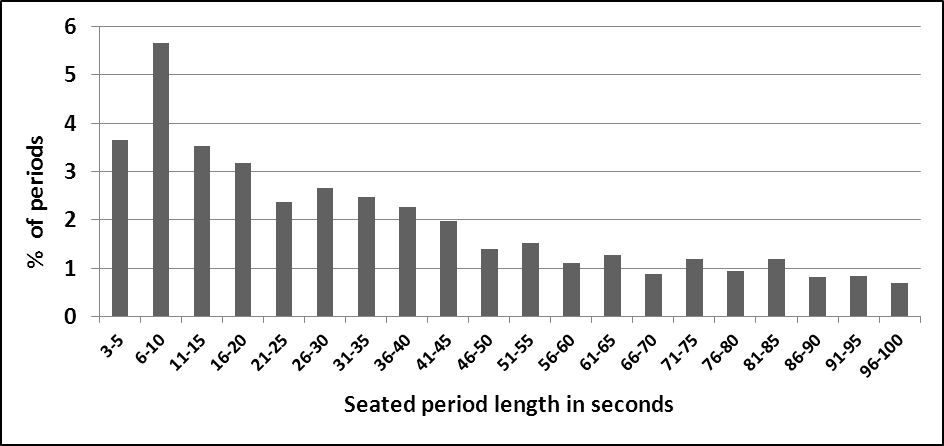


**Figure A4** Proportion of seated periods of 3-100 seconds duration in % of all 4218 recorded seated periods, stratified by period length among 140 call centre operators. Detail of Figure 3 in main file.

*
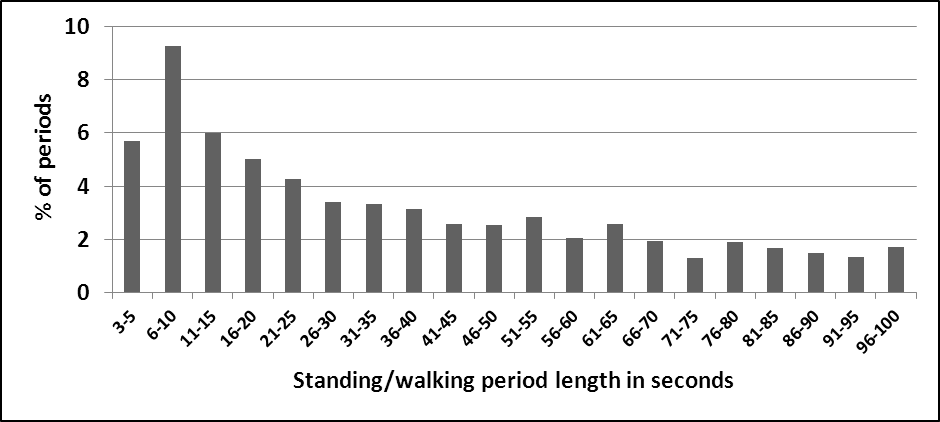
*

**Figure A5** Proportion of standing/walking periods of 3-100 seconds duration in % of all 4357 recorded standing/walking periods, stratified by period length among 140 call centre operators. Detail of Figure 4 in main file.

**Table A1**  Self-reported age, seniority at present company, height, weight and calculated body mass index (BMI) for the CC operators studied at the 16 call centres; in total and amongst male and female call centre operators.

|  |  |  |  | **Difference males-females** | | |
| --- | --- | --- | --- | --- | --- | --- |
|  | **All** | **Males** | **Females** | **Difference** | **p-value^1^** | |
| **Number** | 140 | 43 | 97 |  |  | |
| **Age (years)** | | | | | | |
| **Mean** | 34.4 | 31.0 | 35.9 | -4.9 | | **0.011** |
| **Median** | 33.0 | 29.0 | 35.0 | -6.0 | |  |
| **sd** | 10.6 | 8.8 | 11.1 |  | |  |
| **Range** | 18-62 | 20-53 | 18-62 |  | |  |
| **Seniority at company (years)** | | | | | | |
| **Mean** | 3.54 | 2.78 | 3.84 | -1.06 | | 0.599* |
| **Median** | 2.08 | 1.83 | 2.13 | -0.30 | |  |
| **sd** | 4.36 | 2.49 | 4.90 |  | |  |
| **Range** | 0.17-25.8 | 0.17-9.08 | 0.17-25.8 |  | |  |
| **Height (m)** | | | | | | |
| **Mean** | 1.71 | 1.79 | 1.67 | 0.12 | | **<0.001** |
| **Median** | 1.70 | 1.80 | 1.68 | 0.12 | |  |
| **sd** | 0.085 | 0.081 | 0.058 |  | |  |
| **Range** | 1.53-1.96 | 1.55-1.96 | 1.53-1.81 |  | |  |
| **Weight (kg)** | | | | | | |
| **Mean** | 71.4 | 81.5 | 67.3 | 14.2 | | **<0.001** |
| **Median** | 70.0 | 82.0 | 65.0 | 17.0 | |  |
| **sd** | 13.5 | 10.5 | 12.3 |  | |  |
| **Range** | 47-111 | 60-111 | 47-107 |  | |  |
| **BMI (kg/m^2^)** | | | | | | |
| **Mean** | 24.4 | 25.5 | 24.0 | 1.6 | | **0.016** |
| **Median** | 24.0 | 25.2 | 23.4 | 1.8 | |  |
| **sd** | 3.77 | 2.86 | 4.01 |  | |  |
| **Range** | 16.2-37.7 | 20.1-30.5 | 16.2-37.7 |  | |  |

^1^ Two-tailed t-tests or Mann-Whitney test (*) if significant deviation from normal distribution. Boldface p-values indicate a significant difference (p<0.05).
